# Clinical presentation and outcomes of care in adults with diabetic ketoacidosis pre-COVID-19 and during-COVID-19 at a tertiary, referral hospital in Nairobi, Kenya

**DOI:** 10.1186/s12902-024-01610-8

**Published:** 2024-07-26

**Authors:** Sairabanu Sokwalla, Jasmit Shah, Sangeeta Chauhan, Reena Shah, Salim Surani, Erick Njenga, Nancy Kunyiha

**Affiliations:** 1 Aga Khan University, Nairobi, Kenya; 2 Brain and Mind Institute, Nairobi, Kenya; 3 Texas A&M University, Texas, USA; 4 Mayo Clinic, Rochester, MN USA

**Keywords:** Diabetes ketoacidosis, DKA, Mortality, COVID-19, Precipitants, Aga Khan University Hospital.

## Abstract

**Background:**

Prognosis of DKA has improved over time with the availability of evidence-based protocols and resources. However, in Kenya, there are limited resources for the appropriate diagnosis and management of DKA, mostly limited to tertiary-level referral facilities. This study aimed to review the clinical presentation, management, and outcomes of adult patients admitted with DKA and assess differences in these parameters before and during the COVID-19 pandemic.

**Methods:**

This was a retrospective study of DKA admissions from January 2017 to December 2021. Patient data were retrieved from the medical records department using ICD-10 codes, and individual details were abstracted on clinical presentation, management, and outcomes of DKA. Comparisons were made between pre-COVID-19 and during COVID-19 durations.

**Results:**

150 patients admitted with DKA were included (*n* = 48 pre- COVID-19, *n* = 102 during COVID-19 (*n* = 23 COVID-19 positive, *n* = 79 COVID-19 negative)). Median age was 47 years (IQR 33.0, 59.0), median HbA1C was 12.4% [IQR 10.8, 14.6]), and most patients had severe DKA (46%). Most common DKA precipitants were infections (40.7%), newly diagnosed diabetes (33.3%) and missed medication (25.3%). There was a significant difference in pulmonary infections as a DKA precipitant, between the pre- COVID and during COVID-19 pandemic (21.6% during COVID-19 versus 6.3% pre- COVID-19; *p* = 0.012). Median total insulin dose used was 110.0 units [IQR 76.0, 173.0], and a 100% of patients received basal insulin. Median length of hospital stay was 4.0 days [IQR 3.0, 6.0] and time to DKA resolution was 30.0 h [IQR 24.0, 48.0]. There were 2 deaths (1.3%), none directly attributable to DKA. Severity of DKA significantly differed between pre- COVID-19, COVID-19 positive and COVID-19 negative DKA (52.2% of COVID-19 positive had moderate DKA compared to 26.6% of COVID-19 negative and 22.9% of Pre-COVID-19 (*p* = 0.006)).

**Conclusion:**

Even in developing regions, good outcomes can be achieved with the appropriate facilities for DKA management. Clinician and patient education is necessary to ensure early detection and prompt referral to avoid patients presenting with severe DKA. Exploratory studies are needed to assess reasons for prolonged time to DKA resolution found in this study.

## Introduction

Diabetes is a major health issue and one of the fastest-growing health emergencies worldwide. It is estimated that globally, in 2021, 537 million adults (between the ages of 20–79 years) had diabetes, projected to increase to 643 million by 2030 and 783 million by 2045 [1]. In Africa, an estimated 24 million adults are living with diabetes, and this is expected to increase by 134% by the year 2045 [1]. The estimated prevalence of diabetes in Kenya is currently at 3%, translating to 821,000 adults [1] and this is deemed to be a modest proportion of the actual numbers, given a lack of country-wide epidemiological data [1]. Regional prevalence ranges from urban and rural Kenya found overall prevalence rates of Diabetes at 3.5-5%, with the majority in urban areas [2].

Type 2 diabetes (T2D) is the most common type of diabetes, constituting 90% of diabetes worldwide. Type 1 diabetes (T1D) predominantly affects children and adolescents (over 1.2 million, 54% under 15 years of age). There is limited data on the prevalence of T1D in adults from low and middle-income countries. The Centre for Disease Control reports that 5.8% of all United States adults (over the age of 20 years) diagnosed with diabetes had both a diagnosis of T1D and were using insulin. 10% of all adults with diabetes were using insulin within one year of diabetes diagnosis [3].

Diabetes Ketoacidosis (DKA) is a potentially life-threatening diabetic emergency, predominantly presenting in patients with T1D, either newly diagnosed or precipitated in pre-existing T1D by numerous precipitants, including infection, dehydration, and missed/ inadequate insulin doses, amongst others. DKA is typically marked by acidosis, ketosis, and usually hyperglycemia [4–7]. However, individuals with T2D can present with DKA at diagnosis as seen in ‘Ketosis prone T2D’, which more frequently occurs in Sub- Saharan Africans, Afro-Caribbean or Hispanic descendants [8, 9]. Individuals with T2D can also develop DKA during the course of disease, particularly those with lean T2D as noted by Kibirige et al. [10], and those with long- standing T2D [11].

In children diagnosed with T1D, pooled results from 13 studies showed that 29.9% of them had DKA at diagnosis [12]. A systematic literature review assessing the incidence and prevalence of DKA in adults with T1D reported a range of 0–56 per 1000 person-years (PYs), with one study reporting an incidence of 263 per 1000 PYs. The prevalence of DKA decreased with increasing age, and predisposing patient characteristics for increased DKA risk included: poor glycemic control, depression/psychiatric symptoms, lower socioeconomic status, and female gender [13].

A systematic review analyzing risk factors for adverse outcomes in adults and children with hyperglycemia presenting to the emergency department found that age (< 25 years and > 65 years), lower socio-economic status, use of insulin, complicated diabetes (micro-vascular and macro-vascular complications), recurrent emergency department visits/ hospitalizations, poor .drug adherence, and sepsis were associated with worse outcomes for both DKA and Hyperosmolar Hyperglycemic state (HHS). Patient receiving follow-up and management with a multidisciplinary diabetes team was associated with better outcomes [14].

Early recognition and prompt treatment are crucial in DKA management to avert prolonged hospital stays and increased mortality [15]. Data looking at trends of DKA in the US over a 15-year period revealed that although over the last six years, there was an increase in hospitalization for DKA at an annual rate of 6.3%, the in-hospital mortality from DKA decreased significantly from 1.1 to 0.4% [16]. However, in budget-restricted resource settings, particularly in the developing world, the case-fatality rates for DKA are higher. A recent study conducted over a 5-year duration at the Kenyatta National Hospital, a tertiary referral facility in Nairobi, Kenya, found a mortality rate of 6.9% in children aged 0–18 years presenting with DKA [17].

During the COVID-19 pandemic, an increase is suspected in incidence, severity, and mortality due to DKA due to multiple factors, including the potential effect of the Severe Acute Respiratory Syndrome Coronavirus 2 (SARS-CoV-2) on the pancreatic beta cells, leading to a diabetogenic effect, inflammation, and hyperglycemia due to steroid use as part of the management of moderate-severe COVID-19 pneumonia [18]. Two meta- analysis have also linked COVID-19 with new-onset hyperglycemia and diabetes due to a bi-directional relationship [19, 20].

Among 5029 patients admitted over eight months (February 2020 to September 2020) with DKA in the US, managed using the computerized insulin infusion protocol (CII), 4% had COVID-19 disease. Patients with COVID-19 were older and had higher BMI than those without COVID-19. Older patients with COVID-19 (> 65 years) were more likely to have cardiovascular diseases and other diabetic complications than younger patients. The hospital mortality rate for those with COVID-19 was 30% compared to 5% for those without COVID-19. The mortality rate for patients older than 65 years with COVID-19 was 45% versus those without COVID-19. Similar discrepancies were reported for those below 45 years of age (19% with COVID-19 versus 2% in non- COVID-19). Additionally, patients with COVID-19 had a longer duration of stay with higher insulin requirements on the CII [21].

Other than directly increasing complications related to diabetes in patients with COVID-19, challenges in managing DKA have been reported, particularly in resource-limited settings where protocols are not in place and where there is no or limited access to computerized insulin infusions. The frequency of monitoring blood glucose and insulin/ fluid dose adjustment is restricted to reduce healthcare provider exposure and preserve personal protective equipment. Thus, alternative methods, including the use of subcutaneous insulin injections, have been adopted in some facilities [22, 23].

Optimal treatment of DKA requires the use of evidence-based integrated care pathways/ protocols, which could be from international guidelines or local/ institutional protocols/ algorithms [5]. A recent audit of the management of DKA at the Kenyatta National Hospital found that prompt DKA management according to guidelines was not observed, with delays in patient review and initiation of management by doctors, and an all-cause in-hospital mortality rate of 11.9% at two weeks [24].

There is limited data on DKA’s clinical presentation, management, and outcomes, particularly in adults at private, tertiary, teaching, and referral facilities in Kenya. This study aimed to explain the clinical presentation, management, and outcome of patients admitted with a diagnosis of DKA at a private, tertiary, teaching and referral facility, the Aga Khan University Hospital, Nairobi, and determine the differences in these parameters pre-COVID-19 and during the COVID-19 pandemic.

## Methodology

A 5-year (2017–2021) retrospective audit of hospital-admitted DKA patients at the Aga Khan University Hospital, Nairobi, a private tertiary hospital, was performed. Comparisons were made between pre-COVID-19 (January 2017 to February 2020) and during the COVID-19 pandemic (March 2020 to December 2021).

We included all adult (> 18 years’ age) admissions with a DKA diagnosis for analysis. We used the American Diabetes Association (ADA) diagnostic and severity classification criteria for DKA diagnosis and classification [7]. Mild DKA included alert patients with arterial pH 7.25–7.3, serum bicarbonate 15–18 mEq/l, and serum ketones positive, moderate DKA included alert/drowsy individuals with pH 7-7.25, bicarbonate 10- <15 mEq/l and anion gap above 12 and severe DKA included patients with stupor/coma, PH < 7, bicarbonate < 10 mEq/l and anion gap > 12 [7]. Euglycemic DKA was defined as DKA without significant hyperglycemia (plasma glucose ≤ 11 mmol/l and the presence of ketonemia and/or ketonuria. Resolution of DKA was defined as pH > 7.3, bicarbonate > 15 mmol/L, and blood ketone level < 0.6 mmol/L.

COVID-19 was diagnosed using SARS- COV2 PCR testing of nasal swab samples.

Type of diabetes was based on a file diagnosis, which was documented by Endocrinologist/s after appropriate investigations, including OGTT for GDM and positive autoantibodies (Glutamic acid decarboxylase and/or islet cell antibodies) for T1D and LADA as clinically indicated.

The protocol used for DKA management during the study period was the American Diabetes Association recommended pathway [25].

This retrospective review was carried out after receiving ethical approval from the Institutional Scientific and Ethics Review Committee at the Aga Khan University, Nairobi, and the National Commission for Science, Technology, and Innovation (NACOSTI). All methods were performed in accordance with the relevant guidelines and regulations and in line with the Declaration of Helsinki.

All files for patients between January 2017 to December 2021 were retrieved from the medical records department based on the ICD-10 coding and using the hospital identification numbers. We collected data on clinical presentation, type of diabetes, precipitating factors and severity of DKA, medication use, COVID-19 status and severity, laboratory data on arrival and discharge/ death from critical care, DKA treatment details, length of hospital stays, and mortality rate were collected, de-identified, cleaned and analyzed.

Summary statistics were presented as frequencies and percentages for categorical data and medians and interquartile (IQR) for continuous data. Differences between groups were conducted using univariate analysis, where the Fishers Exact test was used for categorical data, and Kruskal Wallis test was used for continuous data. A *p*-value of less than 0.05 was considered statistically significant.

## Results

A total of 150 patients were included in the analysis. The median age was 47.0 years (IQR: 33.0–59.0), and 60% of the patients were males. The most common comorbidity was hypertension (20%), followed by dyslipidemia (4.0%).

### Admission parameters

**Fig. 1:**
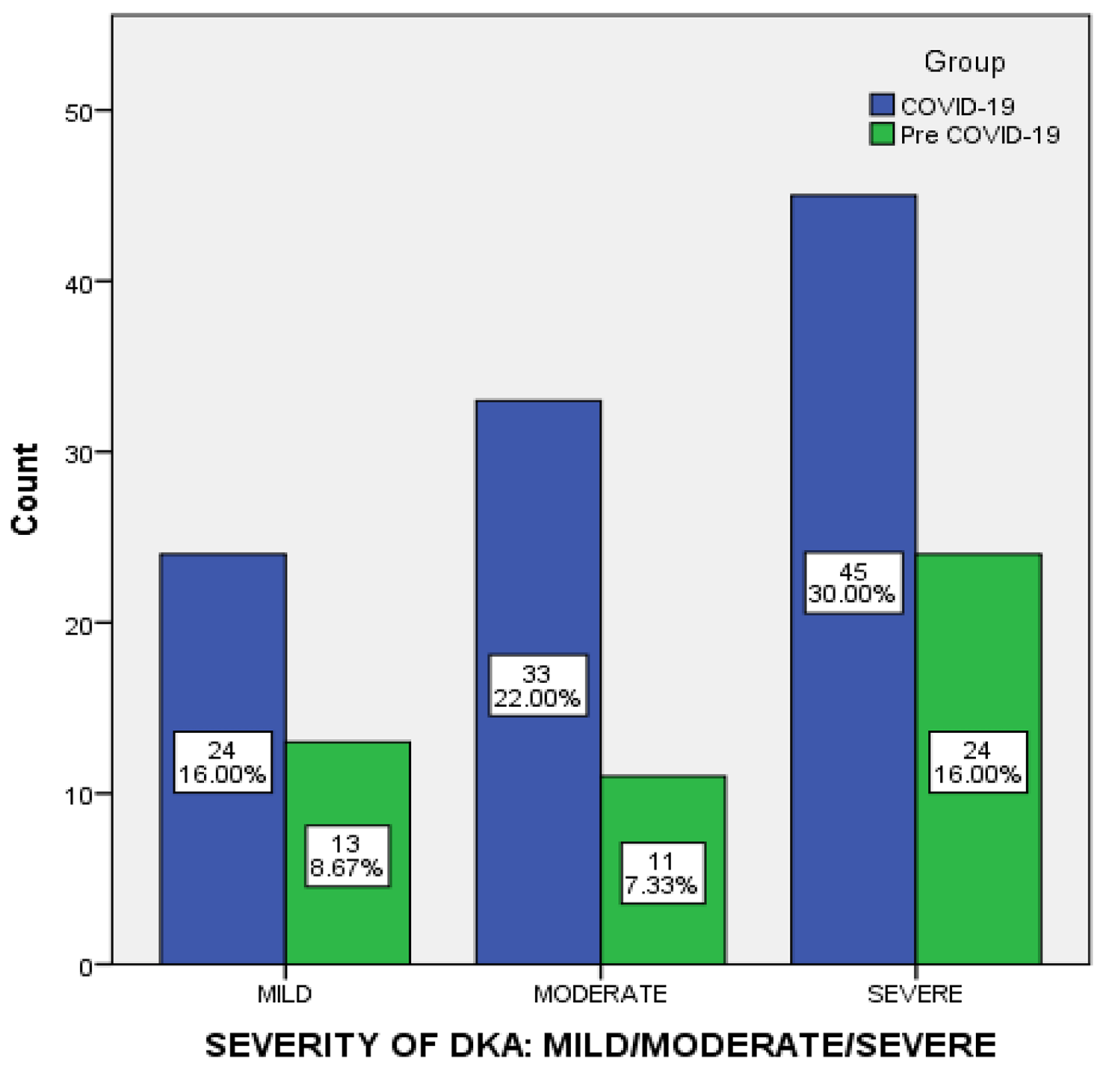
Comparison of Severity of DKA between Pre- COVID-19 and during the COVID-19 Pandemic

Overall, 24.7% of the patients were classified as mild DKA, 29.3% as moderate DKA, and 46.0% as severe DKA (Table 1; Fig. 1). Known diabetes was reported in 60% of the patients, Type 2 diabetes (70%) being the most common type of diabetes, and 55.6% reported taking insulin. The majority of the participants with known diabetes had a diabetes duration of greater than 1 year (52.7%). Almost a third of the patients presented with either osmotic symptoms (10%) or altered levels of consciousness (12.7%).

**Fig. 2:**
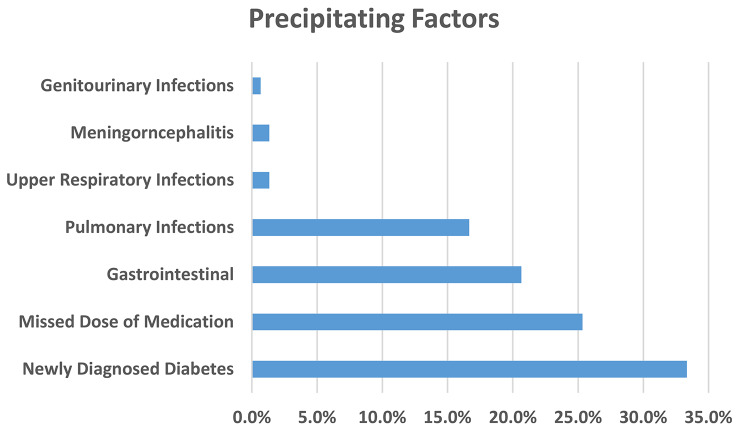
Precipitating Factors of DKA

The most common DKA precipitants were infections (40.7%), newly diagnosed diabetes (33.3%) and missed doses of medications (25.3%). 60% reported missing a dose of medication, where 52.0% reported missing a dose of insulin in the past week, and 16.7% had reported missing a dose of non-insulin medication in the past week (Table 1; Fig. 2). Only 18.0% had reported a prior history of DKA admission. Table 1 summarizes the descriptive analysis of the variables.

**Table 1:** Sociodemographic, clinical, and biochemical characteristics of the participants (*n* = 150)

Age (years) (median [IQR])		47.0 [33.0, 59.0]	
		Frequency	Percentage %
Gender	Females	60	40.0%
	Males	90	60.0%
Comorbidities (*n* = 150)	Hypertension	30	20.0%
	Dyslipidemia	6	4.0%
	HIV	1	0.7%
	Stroke	3	2.0%
	Heart Disease	1	0.7%
	CKD	3	2.0%
	CGD	2	1.3%
Known Diabetes (*n* = 150)	Yes	90	60.0%
	No	60	40.0%
Type of Known Diabetes (*n* = 90)	Type1	26	28.9%
	Type2	63	70.0%
	GDM	1	1.1%
	LADA	1	1.1%
Insulin (*n* = 90)	Yes	50	55.6%
	No	40	44.4%
Medications (*n* = 90)	Glargine (Lantus)	28	31.1%
	Lispro (Humalog)	16	17.8%
	Aspart (Novorapid)	13	14.4%
	Glulisine (Apidra)	1	1.1%
	Analog Mixed (Novomix, Humalog Mix)	3	3.3%
	Human Mixed	14	15.6%
	Others	4	4.4%
	Metformin	23	25.6%
	Sitagliptin	16	17.8%
	Vildagliptin	3	3.3%
	Linagliptin	1	1.1%
	Dapagliflozin	1	1.1%
	Empagliflozin	4	4.4%
	Pioglitazone	2	2.2%
	Glibenclamide	1	1.1%
	Gliclazide	2	2.2%
	Glimerpiride	3	3.3%
	Glipizide	1	1.1%
Duration of Diabetes	Less than 1 month	5	5.6%
	1 month to 1 year	6	6.7%
	More than 1 year	79	87.8%
Missed dose of medication	Yes	54	60.0%
	No	36	40.0%
Missed dose of insulin in the past week	Yes	26	52.0%
	No	24	48.0%
Missed dose of non-insulin in the past 1 week	Yes	15	16.7%
	No	75	83.3%
Prior History of DKA Admission	Yes	27	18.0%
	No	123	82.0%
COVID Status	Positive	23	15.3%
	Negative	79	52.7%
	Unknown	48	32.0%
Presenting Symptoms	Osmotic Symptoms	15	10.0%
	Impaired Level of Consciousness	19	12.7%
	Kussmaul Respiration	1	0.7%
	Stroke	2	1.3%
	Myocardial Infarction	1	0.7%
Precipitating Factors	Missed Dose of Medication	38	25.3%
	Newly Diagnosed Diabetes	50	33.3%
	Pulmonary Infections	25	16.7%
	Genitourinary Infections	1	0.7%
	Upper Respiratory Infections	2	1.3%
	Gastrointestinal	31	20.7%
	Meningoencephalitis	2	1.3%
Severity of DKA	Mild	37	24.7%
	Moderate	44	29.3%
	Severe	69	46.0%
**Markers at Admission**			
Blood Glucose (MMOL/L) (*n* = 124)		22.0 [17.6, 27.0]	
*Blood Glucose > 33 MMOL/L (* *n* * = 150)*		26 (17.3%)	
Plasma Ketones (MMOL/L) (*n* = 125)		3.8 [2.6, 5.7]	
*Plasma Ketones > 8 MMOL/L (* *n* * = 145)*		20 (13.8%)	
PH (*n* = 147)		7.3 [7.2, 7.5]	
PCO_2_ (MMHG) (*n* = 148)		29.0 [21.0, 35.0]	
PAO_2_ (MMHG) (*n* = 148)		58.0 [39.0, 76.5]	
Lactate Levels (MMOL/L) (*n* = 148)		1.9 [1.4, 3.0]	
WBC (10^9/L) (*n* = 148)		9.5 [6.3, 12.9]	
Neutrophils (%) (*n* = 66)		80.0 [70.0, 88.0]	
Lymphocytes (%) (*n* = 3)		46.0 [37.2, 46.0]	
Sodium (MMOL/L) (*n* = 149)		134.0 [131.0, 139.0]	
Potassium (MMOL/L)		4.4 [3.9, 5.0]	
Urea (MMOL/L) (*n* = 149)		5.1 [3.6, 8.4]	
Creatinine (MICG/DL)		92.0 [79.0, 125.0]	
HBAIC (%) (*n* = 148)		12.4 [10.8, 14.6]	
CRP (*n* = 132)		31.0 [6.0, 69.5]	
**Markers at Discharge**			
Blood Glucose (MMOL/L)(*n* = 149)		10.2 [7.5, 12.7]	
Plasma Ketones MMOL/L(*n* = 143)		0.3 [0.2, 0.5]	
PH (*n* = 146)		7.4 [7.4, 7.4]	
PCO2 (MMHG)(*n* = 146)		33.7 [30.0, 38.0]	
PAO2 (MMHG) (*n* = 146)		82.0 [71.0, 97.0]	
Lactate Levels (MMOL/L) (*n* = 144)		1.1 [0.8, 1.6]	
WBC (10^9/L)		6.4 [5.2, 8.8]	
Neutrophils (%) (*n* = 23)		53.0 [43.0, 71.0]	
Lymphocytes (*n* = 1)		44.0 [44.0, 44.0]	
Sodium (MMOL/L)		138.0 [136.0, 142.0]	
Potassium (MMOL/L)		3.9 [3.5, 4.5]	
Urea (MMOL/L) (*n* = 149)		3.6 [2.3, 5.2]	
Creatinine (MICG/DL)		74.0 [60.0, 89.0]	
CRP (MG/L) (*n* = 128)		12.5 [4.0, 46.5]	

*Continuous data were presented with median and IQR in parenthesis

Table 1 also summarizes the lab markers at admission and at discharge. As noted, most patients admitted with a diagnosis of DKA had hyperglycemia, with a mean blood glucose of 22 mmol/l, and 26 (17.3%) had un-recordable high (> 33 mmol/l) capillary glucose levels. The average point-of-care ketone value was 3.8 mmol/l, with almost 14% having un-recordable high levels (> 8 mmol/l). The mean PH at admission was mildly low at 7.3 [IQR 7.2, 7.5]. Neutrophilia was the most predominant blood count abnormality (80.0% [IQR 70.0, 88.0]), and the mean CRP was elevated at 31 mg/l. The average HbA1C was high at 12.4% [IQR 10.8, 14.6]. On average, patients admitted with DKA had normal renal functions at admission.

### Treatment of DKA

The majority of the patients were treated with normal saline (98.0%), followed by Dextrose Normal Saline (DNS) (70.0%) and ringers’ lactate (62.0%). All patients were treated with Glargine (100%) insulin and insulin infusion using short acting insulins (human or analog). Potassium supplementation was reported in 85.3% and 98.7% had deep venous thrombosis (DVT) prophylaxis.

### Outcomes of DKA

The median length of hospital stay was 4 days (IQR 3.0,6.0), and critical care stay was 48 h (IQR 1.0–3.0). The median total insulin dose used for DKA resolution was 110 units, including both basal and short-acting insulins, and time to DKA resolution was 30 h (IQR 24,48).

Only 2 patients (1.3%) were reported to have died (Table 2).

One of the deaths was due to cardiorespiratory arrest caused by severe COVID-19 pneumonia and occurred within less than 24 h of admission. However, DKA in this patient had resolved within 6 h of hospitalization to ICU. The second death was in a patient with a massive pulmonary embolism, intracranial hemorrhage with intra-ventricular spill, and severe sepsis with multi-organ failure. In this patient, DKA had resolved within 24 h of admission, and death occurred on day 30 of admission. Therefore, in this study, there was no mortality attributable directly to DKA over the 5-year period.

**Table 2:** Outcomes of DKA admissions

Length of Hospital Stay (Days)*			4.0 [3.0, 6.0]
Length of Critical Care Stay (Days) (*n* = 131)*			2.0 [1.0, 3.0]
Total Insulin Dose used for DKA Resolution (*n* = 149)*			110.0 [76.0, 173.0]
Time to DKA Resolution from Presentation to Casualty*			30.0 [24.0, 48.0]
		Frequency	Percentage (%)
Outcome	Death	2	1.3%
	Discharge	148	98.7%
DKA Complications	Cerebral Edema	1	0.7%
	Shock	4	2.7%
	Acute Kidney Injury	22	14.7%
	DVT	1	0.7%
	ARDS	1	0.7%
	Liver Disease	1	0.7%

*Data were presented with median and IQR in parenthesis

### DKA and COVID-19 infection

Based on admissions during the COVID-19 pandemic (March 2020 to December 2021) (*n* = 102), 22.5% (*n* = 23) tested positive, and 77.5% (*n* = 79) tested negative for COVID-19 infection.

The severity of DKA was associated with COVID-19 status; 52.2% of the positive COVID-19 had moderate DKA compared to only 26.6% of the negative COVID-19 and 22.9% of the Pre-COVID-19 (*p* = 0.006). Table 3 shows the association between COVID-19 status with DKA and diabetes.

On comparing the pre-COVID-19 and during-COVID-19 data, the most common precipitating factor was infection and did not differ between the two, but pulmonary infection was significantly more in DKA patients during the COVID-19 pandemic compared to pre-COVID-19 (21.6% versus 6.3%, *p* = 0.012). Other precipitants did not differ between groups. Likewise, the severity of DKA and known diabetes status were not significantly different between the groups.

Outcomes of DKA, including mortality, the total dose of insulin used, time to DKA resolution, and length of hospital stay, did not differ significantly between pre-COVID-19 and during the COVID-19 pandemic (Table 4).

**Table 3:** Relationship of COVID-19 Status with Diabetes Diagnosis and DKA Severity

		COVID-19 Status						*P* Value
		Positive		Negative		Pre-COVID		
		Frequency	Percentage (%)	Frequency	Percentage (%)	Frequency	Percentage (%)	
Known Diabetes	No	6	26.1%	33	41.8%	21	43.8%	0.348
	Yes	17	73.9%	46	58.2%	27	56.3%	
Severity of DKA	Mild	8	34.8%	16	20.3%	13	27.1%	**0.006**
	Moderate	12	52.2%	21	26.6%	11	22.9%	
	Severe	3	13.0%	42	53.2%	24	50.0%	

***Bold*** *- statistically significant value*

**Table 4:** Comparison of Presentation and Outcomes of DKA pre-COVID-19 and During the COVID-19 Pandemic

		COVID-19		Pre COVID		*P* Value
		Frequency	Percentage %	Frequency	Percentage %	
Severity DKA	Mild	24	23.5%	13	27.1%	0.496
	Moderate	33	32.4%	11	22.9%	
	Severe	45	44.1%	24	50.0%	
Precipitating Factors	Missed Dose of Medication	26	25.5%	12	25.0%	0.949
	Newly Diagnosed Diabetes	35	34.3%	15	31.3%	0.710
	**Pulmonary Infections**	**22**	**21.6%**	**3**	**6.3%**	**0.012**
	Genitourinary Infections	1	1.0%	0	0.0%	0.379
	Upper Respiratory Infections	2	2.0%	0	0.0%	0.212
	Gastrointestinal	20	19.6%	11	22.9%	0.643
	Meningoencephalitis	1	1.0%	1	2.1%	0.596
Known Diabetes	No	39	38.2%	21	43.8%	0.520
	Yes	63	61.8%	27	56.3%	
Total Insulin Dose Used for DKA Resolution*		109.0 [77.0, 173.0]		116.0 [66.0, 173.0		0.736
Time to DKA Resolution*		32.0 [24.0, 48.0]		30.0 [24.0, 48.0]		0.400
Length of Hospital Stay*		4.0 [3.0, 6.0]		4.0 [3.0, 6.0]		0.754

*Data were presented with median and IQR in parenthesis; ***Bold*** *- statistically significant value*

## Discussion

Management of DKA requires early identification through a high index of suspicion by taking a thorough yet targeted history and clinical examination and utilization of appropriate diagnostic tools, including blood glucose, ketone, blood gas, and electrolyte measurement, alongside other supportive tests to identify predisposing/ precipitating factors, comorbidities and complications [26]. Having access to multidisciplinary care through diabetes teams and the availability of well-laid-out pathways for DKA management is also highly recommended, in addition to critical care and monitoring support [26].

At the Aga Khan University Hospital, a tertiary level, private teaching, and referral facility, there is access to most of the required services and resources spite it being in a developing global region. This study found that amongst patients admitted with DKA over a 5-year period (2017–2021), including 2 years of the COVID-19 pandemic (2020 to 2021), the majority had type 2 diabetes, were males, and had missed their doses of medications in the previous one week. Infection was found to be the most likely precipitant of DKA in our patients (predominantly gastrointestinal and pulmonary), followed by newly diagnosed diabetes and missed doses of medication. Mortality in our study was low at 1.3% (2 patients), and both patients who died had resolved DKA at the time of death and had underlying fatal conditions. Therefore we had no mortality directly attributable to DKA.

Desse et al. assessed the predictors and treatment outcomes of hyperglycemic emergencies at a public University hospital in Ethiopia and found that the majority of patients were male, and over 90% had DKA, though 64% had T1D [27]. Our study also found a male predominance, but a higher proportion of our patients known to have diabetes had T2D (70%). In a similar study conducted in South Africa, there was no significant gender difference, and 60% of patients with DKA had T1D, similar to the Ethiopian study and a more recent study at the Kenyatta National Hospital in Kenya [24, 27, 28]. In all these studies, the lower cut-off age value was less at 14, 15, and 13 years in the South African, Ethiopian, and Kenyan studies, respectively, compared to 18 years in our study [24, 27, 28]. The mean age was higher in our study (47 years), explaining the higher proportion of patients having T2D versus T1D. A 14-year retrospective study from Bangkok, Thailand, had a higher mean age of patients with DKA (47.4 ± 20.4 years) and found a higher proportion of patients with T2D, like our study. They, however, found a higher female predominance (61.5%) [29].

About a 10th of the patients with DKA in this study presented with osmotic symptoms, and almost one in five had altered levels of consciousness. Desse et al. found that the most common presenting symptoms in Ethiopia were polyuria and polydipsia, similar to findings from Nigeria and Libya [27, 30, 31]. However, another Ethiopian study found that abdominal pain and vomiting were the prominent presenting features [32]. In Kenya, an altered level of consciousness was found in over 90% of presentations for DKA in a public tertiary referral facility, with almost a quarter in coma [33]. The majority of our patients presented with severe DKA (46%), and almost a quarter had mild DKA. This was contrary to the study by Desse et al., which found that three-quarters of the patients had mild DKA, with only 5.4% having severe DKA [27]. A study from Thailand in similar settings found that 45.7% of their patients had mild DKA, and severe DKA was found in about a third of their patients [29]. The differences could be explained by later presentations and referrals to our facility and possible lack of early recognition and detection of DKA at primary and secondary level hospitals since similar findings are noted from other studies in Kenya [24, 33]. Another explanation could be the poorer glucose control (high HbAIc) in our study, indicating a lower threshold to DKA and therefore more severe DKA at presentation.

Similar to our study, in their study, Desse et al. found that hyperglycemic emergencies were most precipitated by infections (59%), poor medication compliance (32.3%), and a new diagnosis of Diabetes (23.6%) [27]. However, in their study, urinary tract infections were the predominant infection, followed by pulmonary infections. In our study, gastrointestinal and pulmonary infections were the most common precipitant infections for DKA, similar to a study from Thailand, which found gastrointestinal infections (40%), urinary tract infections (22%), and pulmonary infections (15%) as the most common precipitant infections [29]. In our study, pneumonia as the most common precipitant could be due to COVID-19 pandemic. However, a similar study from Kenya, conducted about 18 years ago in a public teaching and tertiary referral hospital, found that pulmonary and genito-urinary infections were the most common precipitant infections for DKA, similar to a recent study from the same facility [24, 33]. Compared to the previous study from Kenya, the more recent one (2018) found that a new diagnosis of diabetes was a more common precipitant of DKA than infections [24, 33]. Additionally, in a study in China on DKA precipitants in patients with T2D, pulmonary and urinary tract infections were the most common precipitants [34]. In our study, only 1 patient had a genitourinary infection as a DKA precipitant, though the diagnosis was chart based, not on a urine analysis. This could have missed silent/ asymptomatic genitourinary infections. Therefore, we feel that this needs further exploration.

Early recognition and prompt treatment are crucial in DKA management to avert prolonged hospital stays and increased mortality [15]. The mainstay of treatment remains rehydration, early insulin initiation, identification and treatment of precipitants, and management of electrolyte imbalance [26]. In our study, the mean amount of insulin used was 110 units, including both basal and short-acting. We had a 100% uptake of basal insulin during the duration of DKA, which could have contributed to a lower overall use of insulin for DKA resolution compared to other similar studies. The mean amount of insulin used in the study by Desse et al. was 136.85 ± 152.41 units till the resolution of DKA/ HHS, and they did not specify the use of basal insulin [27].

Our study’s most frequently used fluid for DKA management was normal saline, followed by dextrose-saline (DNS) when the sugars were lower than 14 mmol/l. However, of note is that almost two-thirds of our patients received ringers’ lactate at some point during DKA management. The current guidelines still recommend normal saline as the first choice of fluids [26, 35] for DKA management, but there is emerging evidence on the role of balanced crystalloids like Plasmalyte and Ringer’s lactate as the fluid of choice for rehydration in DKA to reduce hyperchloremia and improve renal functions [36–38]. A small, randomized clinical trial conducted in South Africa, comparing Normal Saline with Ringer Lactate for DKA management revealed no difference in time to DKA resolution and that it took longer for glucose normalization with Ringer Lactate compared to Normal Saline [39].

Our study’s average length of hospital stay was 4 days, with a mean of 48 h in critical care, whereas the mean time to DKA resolution (from the presenting time to casualty) was 30 h. The length of hospital stay was slightly more than a similar study from Thailand (3 days) but less than studies from Ethiopia (5 and 6 days) and South Africa (8.9 days) [27–29, 32]. In the United Kingdom, the median length of hospital stay following adherence to the DKA protocol was 2 days, with a median time to DKA resolution of 12 h and 6 min [40]. The median time to DKA resolution in the study from Thailand was lower than in our study and the UK study, at only 8 h, whereas the study from Ethiopia by Desse et al. found a mean time to DKA resolution of 64.38 h, which was longer than in our study. The reason for the relatively long duration of DKA resolution in our study is not clear and could be due to delays in treatment initiation or maintenance, or due to patients presenting in severe DKA, requiring longer time to resolution. However, this needs further exploratory studies.

Data looking at trends of DKA in the US over a 15-year period revealed that although over the last six years, there was an increase in hospitalization for DKA at an annual rate of 6.3%, the in-hospital mortality from DKA decreased significantly from 1.1 to 0.4% [16]. A retrospective study in a tertiary-level facility in Saudi Arabia also found low mortality rates at 1.83% [41].

However, in budget-restricted resource settings, particularly in the developing world, the case-fatality rates for DKA are higher. A recent study conducted over a 5-year duration at the Kenyatta National Hospital, a tertiary referral facility in Nairobi, Kenya, found a mortality rate of 6.9% in children aged 0–18 years presenting with DKA. The average duration of stay in this study was eight days, and factors associated with high mortality were high serum creatinine, decreased urine output, and altered consciousness level [17]. A study conducted at the same facility over 15 years ago, including adults (age over 12 years) admitted with a diagnosis of DKA, found that half of the patients were newly diagnosed with diabetes, and 90% had high HBAIC (> 8%). Patients presented with complicated DKA, having altered levels of consciousness, severe hypotension, and moderate-severe dehydration. The study showed a high mortality rate of 29.8% within 48 h of admission [33] High mortality rates of over 17% were also reported from a rural facility in South Africa [28]. However, a study conducted in a multidisciplinary diabetes center in Thailand found a lower mortality rate of 4.3% but high rates of hypoglycemia and hypokalemia [29].

Overall mortality from hyperglycemic emergencies in the Ethiopian study was 9.8%. High admission creatinine, sepsis, and the presence of comorbidities were associated with high mortality [27].

In this study, despite patients presenting with more severe DKA and having poorly controlled diabetes, the much lower mortality can be attributed to several factors, including the availability of specialist endocrinologists for all DKA admissions, admission and care in high dependency and critical care units, use of a standard DKA pathway, availability of Point of care (POC) glucose, ketone, blood gas monitoring, and good laboratory support.

Almost 80% of patients in our study did not develop any DKA complications. The most common complication in our study was acute kidney injury (AKI) (14%), but at discharge, the mean creatinine was within the target range. Circulatory shock was recorded in 2.7% of our patients. In the study from Thailand, hypokalemia was the most common complication (26%), similar to a study from Saudi Arabia, which also found AKI as a common complication [29, 41]. We did not find hypokalemia as a complication because of the high potassium supplementation in our study and the frequent monitoring of potassium through blood gases and laboratory tests.

Our study included patients before and during the COVID-19 pandemic, both COVID-19-positive and negative patients. Although the numbers of COVID-19-positive patients with DKA were small (23 patients), we attempted to compare DKA severity and outcomes between the three groups. We found that there was no difference in pre-diagnosis of diabetes between the groups, but there was a significant difference in the severity of DKA. More patients who were COVID-19 positive presented with moderate DKA compared to those who were negative for COVID-19 or those in the pre-COVID pandemic, the majority of whom presented with severe DKA. In our study, a comparison between the pre-COVID-19 pandemic and during the COVID-19 pandemic found a significant difference in pneumonia as a DKA precipitant, but there was no statistically significant difference in DKA severity, or DKA outcomes (mortality, units of insulin used, length of hospital stay and time to DKA resolution).

A large retrospective study from the US assessing DKA outcomes pre and during the COVID-19 pandemic (COVID-19 PCR+) included 11 facilities in New York, and they also found that there was no significant difference in the pre-diagnosis of diabetes pre-pandemic and during the COVID-19 pandemic. They did not find significant differences in the severity of DKA between the two periods. However, they did find a significant difference in mortality; 46.3% during the COVID-19 pandemic versus 18% pre-pandemic [42]. However, 2 recent meta- analysis have found a significant association between COVID-19 infection and new onset diabetes; Li et al. found a 1.75 fold increased risk of diabetes and hyperglycemia in those with COVID-19 infections versus those without, and Ssentongo et al. found a 66% increased risk of incident diabetes in those with COVID-19 infection [19, 20].

### Strengths/ limitations

This is the first long-term study, to the best of our knowledge, to be carried out to audit the clinical presentations and outcomes of adult DKA patients in a private tertiary-level referral and teaching facility in Kenya, where adequate resources are available, including monitoring (point-of-care ketone, frequent blood glucose, blood gas, and laboratory support), availability of the recommended nurse-to-patient ratio, appropriate critical care support and sub-specialty care. This is also the first study to assess the clinical presentation and outcomes of DKA in adults during the pre-COVID and COVID-19 pandemic.

Our study has limitations inherent to Retrospective studies and could have had an information bias since the data collected was before the use of a full electronic health record system. Additionally, the diagnosis of the type of diabetes was based on clinical parameters rather than conducting auto-antibody or C-peptide tests.

## Conclusions

This study found a low mortality attributable to DKA and appropriate length of hospital stay for DKA, but the time to DKA resolution was longer than recommended and more patients in our study presented with severe DKA. The outcomes of DKA did not differ significanlty between the COVID-19 pandemic and pre- COVID-19 period. These findings indicate that even in developing regions, good outcomes can be achieved with the appropriate facilities for DKA management. Clinician and patient education is necessary to ensure early detection of DKA and prompt referral to avoid patients presenting with severe DKA. Exploratory studies are needed to assess reasons for prolonged time to DKA resolution found in this study.

## Data Availability

The datasets used and/or analyzed during the current study are available from the corresponding author on reasonable request.

## Declarations

### Author information

Four of the authors (SS, NK, SC, EN) are members of the Kenya Diabetes Study Group, actively engaged in research in diabetes and endocrine conditions in Kenya.

## Abbreviations

T2D: type 2 diabetes mellitus
T1D: type 1 diabetes mellitus
AKUH: Aga Khan University Hospital
AKUHN: Aga Khan University Hospital Nairobi
BMI: Body Mass Index
BP: blood pressure
SBP: systolic blood pressure
DBP: diastolic blood pressure
DKA: diabetic ketoacidosis
HbA1C: glycated hemoglobin
HHS: hyperosmolar hyperglycemic state
Eu-DKA: euglycemic diabetic ketoacidosis
COVID-19: coronavirus disease-2019
SARS-CoV-2: Severe Acute Respiratory Syndrome Coronavirus 2
US: United States of America
CII: Computerized insulin infusion protocol
